# Functional decay in tree community within tropical fragmented landscapes: Effects of landscape-scale forest cover

**DOI:** 10.1371/journal.pone.0175545

**Published:** 2017-04-12

**Authors:** Larissa Rocha-Santos, Maíra Benchimol, Margaret M. Mayfield, Deborah Faria, Michaele S. Pessoa, Daniela C. Talora, Eduardo Mariano-Neto, Eliana Cazetta

**Affiliations:** 1 Graduate Program in Ecology and Biodiversity Conservation, Applied Ecology and Conservation Lab, State University of Santa Cruz, Ilhéus, Bahia, Brazil; 2 School of Biological Sciences, The University of Queensland, St Lucia Campus, Queensland, Australia; 3 Botany department, Biology institute, Federal University of Bahia, Salvador, Bahia, Brazil; Chinese Academy of Forestry, CHINA

## Abstract

As tropical rainforests are cleared, forest remnants are increasingly isolated within agricultural landscapes. Understanding how forest loss impacts on species diversity can, therefore, contribute to identifying the minimum amount of habitat required for biodiversity maintenance in human-modified landscapes. Here, we evaluate how the amount of forest cover, at the landscape scale, affects patterns of species richness, abundance, key functional traits and common taxonomic families of adult trees in twenty Brazilian Atlantic rainforest landscapes. We found that as forest cover decreases, both tree community richness and abundance decline, without exhibiting a threshold. At the family-level, species richness and abundance of the Myrtaceae and Sapotaceae were also negatively impacted by the percent forest remaining at the landscape scale. For functional traits, we found a reduction in shade-tolerant, animal-dispersed and small-seeded species following a decrease in the amount of forest retained in landscapes. These results suggest that the amount of forest in a landscape is driving non-random losses in phylogenetic and functional tree diversity in Brazil’s remaining Atlantic rainforests. Our study highlights potential restraints on the conservation value of Atlantic rainforest remnants in deforested landscapes in the future.

## Introduction

We are currently in the midst of a massive global extinction event [1], with the current wave of biodiversity reduction mainly occurring due to habitat loss and habitat modification [1,2]. For plants in particular, studies have shown that the synergistic effects of habitat loss and fragmentation lead to profound changes in patterns of tree species composition [3,4], richness [5–7], functional [8–11] and phylogenetic diversity [12–15], with landscape configuration and edge effects commonly identified as the key drivers of floristic decay in tropical rainforests.

The amount of forest cover at the landscape-scale is currently considered an important driver of biological change, and a meaningful proxy for habitat loss [16]. The ‘habitat amount hypothesis’ [17], in fact, posits that “patch size” and remnant “isolation” can be replaced by a single predictor variable—the amount of the remnant habitat found across a landscape. Moreover, the fragmentation threshold hypothesis [18,19] suggests that the amount of forest in the landscape is an important driver of species diversity, and that when forest remnants are in landscapes within higher percentages of total overall forest cover (> 50%) they are prone to maintain higher diversity than those in landscapes with less total forest cover. Under this hypothesis, patch size is also thought to contribute to biodiversity maintenance in landscapes with intermediate levels of forest cover (>30 and <50%), whereas below this threshold (30%) an abrupt, non-linear loss of diversity is expected to occur regardless of patch size [19]. Both theories have been accepted by the landscape ecology and conservation communities (but see [20]), leading to the widely held belief that the amount of forest within a landscape is an effective way of measuring habitat loss and fragmentation, which is easier and more widely accessible information than patch size and isolation [17].

Investigating the species extinction thresholds, *i*.*e*., the minimum amount of habitat area at a landscape scale required for a species to persist, has gained prominence in recently proposed conservation management strategies for human modified tropical landscapes. Yet few studies have evaluated the effects of habitat loss on the occurrence of extinction thresholds for plants at family [7,21], or community levels [19]. Theoretical models, have previously fail to predict extinction thresholds for community responses to landscape modification mainly due to the specific functional traits of different species composing it [22,23]. Therefore, more studies are needed to determine if thresholds hold across families and communities or when taking into account species’ functional attributes.

Apart from examining landscape-level thresholds in species richness, several studies have investigated phylogenetic and functional diversity patterns to assess how biological communities respond to different levels of anthropogenic disturbances [13,21,22]. In fact, both approaches are increasingly common due to the growing emphasis on the idea that richness patterns alone can lead to incorrect interpretations of the effects of habitat loss and fragmentation on biodiversity [23–25]. Investigating phylogenetic associations, like families, can improve studies by allowing similar ecological species to be considered together, acting as a proxy for phylogenetic and functional diversity [26].

Ecologists recognize that studying functional traits or functional groups can provide valuable information regarding ecosystem processes, function and evolutionary history in human-altered landscapes [24]. For instance, species with functional traits of habitat-specialists are frequently more negatively impacted by forest loss across landscapes, in contrast to generalist species [19,25]. The disproportionate loss of some plant functional traits in fragmented forest landscapes, like shade-tolerant and large-seeded species [26–28], can have detrimental effects on food availability [29], shelter for native animals [30], and biomass accumulation [31,32]. In tropical forests, late successional plants—mainly shade-tolerant species, including representative families such as Myrtaceae, Sapotaceae and Rubiaceae—are usually negatively affected by forest cover loss, whereas shade-intolerant pioneer families can be positively influenced by forest cover loss [5,15,21]. Despite functional traits and groups have received growing attention in studies of community ecology and conservation biology [33], there is still a lack of information on the functional responses of tree assemblages in relation to forest cover thresholds in fragmented forest landscapes (but see [34]).

Here, we investigated how tree community, families, and functional traits are affected by forest cover reduction in contrasting landscapes across the Brazilian Atlantic rainforest region. Southern Bahia State is home to some represented largest remnants of Atlantic forest in northeastern Brazil and has a recent history of fragmentation (late twentieth century [35]). In this region, we evaluated whether loss of landscape-scale forest cover corresponds to a functional decay (i.e., a collapse in taxonomic and functional diversity) in forest remnant plant assemblages. To do this, we performed floristic surveys at twenty forest remnants found across a gradient of landscape-scale native forest cover, ranging from 3% to 93% forest cover around focal forest remnants. First, we evaluated the similarity of tree species composition among sites. Then, we investigated the relationship between forest cover and tree species richness and abundance, using a model selection approach to evaluate whether these relationships were linearly or non-linear, and in case of non-linear results, we then identified potential “thresholds” of species and functional diversity loss. We further assessed the diversity patterns for the most common tree families in our surveys (Myrtaceae, Sapotaceae, Fabaceae, Rubiaceae, and Lauraceae). Finally, we explored the richness and abundance of species with three key functional tree traits (regeneration strategy, dispersal mode, and seed size). We hypothesized that forest cover amount would be a strong driver of tree community attributes in our study area. Specifically, we expect that: (*i*) highly deforested landscapes are more similar to each other in terms of species composition, following the floristic differentiation hypothesis [36]; (ii) both species richness and abundance is exhibited to reflect a nonlinear pattern and be greater in landscapes with more forest cover, following the predictions from the Habitat Amount Hypothesis [17] and; (*iii*) the most common families will vary in response to a landscape-wide loss of forests according to family specific requirements; (*iv*) species richness and abundance of shade-intolerant, small-seeded and abiotic-dispersed species (seeds dispersed by anemochory or autochory) will decrease in landscapes with more forest cover, due to the prevalence of disturbance-adapted species within these functional traits [5,37]; whereas large-seeded, shade-tolerant and biotic-dispersed species will be less common in landscapes with less forest cover given that such species are known to be vulnerable to forest loss and fragmentation [9,38].

## Methods

### Study area

We conducted this study in twenty forest sites of the Brazilian Atlantic rainforest in southern Bahia (15°0' - 16°0' S and 39°0'– 39°30' W), which harbors one of the highest diversities of wood plant species in the world [39,40]. All sampled forest sites were located across a gradient of landscape-scale forest fragmentation, in tropical lowland rainforests fragments [41], surrounded by a matrix of agroforestry, pastures, rubber, or eucalyptus plantations [42,43]. Sample sites were at least in medium to advanced stages of secondary succession and were variable in size, but only forest sites larger than 3ha were included. Deforestation occurred in all fragments at about the same time, starting in the mid 1980’s, increasing during the cocoa crisis of the 1990’s. The whole study region’s climate is classified as *Af*, hot and moist, without a distinct dry season [44], according to the Koppen classification, and has an annual average temperature of 24°C and rainfall of ~1,500 mm y^-1^ [45].

### Sampling design

We firstly mapped the forest coverage across an area of 3,500 km^2^, between the Jequitinhonha and Contas Rivers. This region is formed by the same soil, topography, and vegetation types [39]. Regions dominated by Montane Forest (mostly in the northwest, at elevations of 500 m a.s.l. or higher) and Restinga Forests (mostly in southeast) were excluded to avoid these distinct vegetation and soil types. Using high-resolution satellite images (RapidEye, with tiles from 2009 to 2010, and QuickBird and WorldView from 2009 to 2011) of this study region, we created a map by visual interpretation of land cover features at a scale of 1:10,000, which is adequate for identifying patches based on the visual inspection of differences in color, texture, shape, location, and context. To characterize each landscape we quantified the percent forest cover around each site from the center of each forest site (using ARCGIS). We considered only mature and secondary native forests in our estimations of forest cover, excluding shade cocoa and rubber plantations. Although we recognize that agroforests can enhance habitat availability and forest connectivity between forest sites [46], they differ even from regrowth forests considerably in our high-resolution images and since only a small number of surveyed sites were embedded within an agroforestry mosaic we excluded these sites. All selected forest sites were classified according to vegetation types following the typologies provided by the Brazilian Institute of Geography and Statistics [47]. Once patches were identified, we delimited them as polygons and digitally mapped them using ArcGIS software.

From the above map we selected all possible forest sites (total of 58), that spanned a range of landscape-scale forest covers and ground-truthed them all. All selected sites were checked to ensure that they were composed of our focal forest type (tropical lowland rainforests) and that no sites were less than 1 km from any other. Ground truthing also involved determining which sites were accessible and for which we could obtain formal consent from land-owners to survey (for details see [25]). Of the sites that fit these criteria post ground truthing, we used a stratified sampling approach to select 20 focal forest sites from across the full range of forest cover classes. We then adopted a patch-landscape approach, in which the response variables were evaluated within forest sites and the landscape variable (here represented by forest cover) were measured within a specific area around each sampling site [48]. The four spatial scales we used to assess landscape forest cover were: 500, 1,000, 1,500 and 2,000 m radii circles, which captured total landscape areas of 78,5 ha, 314 ha, 706,5 ha and 1256 ha, respectively. These scales were chosen based on similar studies from the literature, and because they seem to appropriately account for relevant landscape factors for seed dispersal [49], pollination functions [50] and plant structure [11].

### Floristic surveys and species classification

We established five 4 x 25 m forest-plots (0.01 ha) close to the center of each selected forest remnant. Plots were positioned at least 50 m apart from each other and from the nearest forest edge, whenever possible (85% of the total). In each forest-plot, we measured and identified all live trees (including arboreal palms) with a diameter at breast height (DBH = 1.30m above ground) of ≥ 5 cm and that had at least 10% of the main trunk partially within plot boundaries. Identifications were performed by experienced botanists to the lowest possible taxonomic level, according to the APG III (2009) classification, based on herbaria collections of Brazilian Institutes located in the Bahia state (CEPEC/Ceplac, UESC and UFBA).

For all surveyed species, we obtained trait values for three categorical functional traits: regeneration strategy, dispersal mode and seed size. Trait data were gathered from published literature [51–53] and authors’ experience with species in the study region. We selected these traits given their known association with important life-history traits in tropical forest systems. Specifically, regeneration strategy is associated with tree recruitment, establishment and survival [3,54]; dispersal mode is important for the maintenance and spreading of viable populations [55]; and seed size is correlated to a range of important processes including seed dispersal, germination success and seedling survival [56,57]. To each species that we could identify to at least the family level and with available information, we assigned states for each functional trait. Even though some family traits are labile, we were able to assign traits in some specific cases in which the species were not identified, but for which fruits were collected, or when the whole family presented the same trait in all Atlantic forest biome (for instance, in Atlantic forest all Myrtaceae species are biotically-dispersed [58,59]; all Sapotaceae species are shade-tolerant [21]; and Melastomataceae species are typically shade-intolerant [12,42,60,61]). For regeneration strategy we classified species as shade-tolerant or shade-intolerant [62]. For dispersal mode, species were classified as vertebrate biotic-dispersed or abiotic-dispersed (seeds dispersed by anemochory or autochory- free fall or propelled explosively by fruit opening). For seed size, data were gathered as a continuous trait and then species were classified as having small seeds (seeds smaller or equal to 1.2 cm of diameter) or large seeds (seeds larger than 1.2 cm diameter). This criterion considers the patterns of seed consumption by frugivores in tropical forests, in which seeds smaller than 1.2 cm are eaten by small birds (including those from Turdidae family), and above this size by large-bodied bird and mammal species [32,63,64]. Based on overall representativeness (number of species) we conducted analyses considering the most common families, separately: Fabaceae, Myrtaceae, Sapotaceae, Rubiaceae, and Lauraceae. Fabaceae is often the most abundant family in floristic surveys across the Atlantic Forest region [45,60,65]. Myrtaceae, Sapotaceae and Rubiaceae are composed of shade-tolerant species typical of most preserved areas in the Atlantic Forest [7,21,60,66], are of great importance as food resources for native animals [66–68], and are among the most diverse families in this part of Brazil [45,53,65]. Lauraceae is abundant and highly diverse in the Atlantic Forest region [45,60,61], also representing important animal food resources [66].

### Ethics statement

Field work was carried out on private properties with permits from each landowner. We did not perform any other activities that required specific government permissions.

### Data analysis

To assess the relationship between forest cover and tree community, we started by comparing the four selected landscape scales to identify the scale that best captured variation in diversity in this study. To do this, we used the software Focus [69] to assess Spearman’s rank test (r) at each of our four pre-established landscape scales (500, 1,000, 1,500 and 2,000 m radii) and determine which scale ranks highest for each response variable (richness and abundance patterns, overall and for each functional trait and family). We used the total number of individuals per fragment as a measure of abundance. We detected that 1,000 m scale showed the highest coefficient values for the majority of richness (86%) and abundance (57%) models, so we used this radius for forest cover estimates for all subsequent analyses.

To investigate the potential spatial dependence on the patterns of species richness and abundance, we applied a semivariogram (see [70]), a spatial technique widely applied to examine and identify spatial dependency and the spatial structure of that dependency. We included spatial covariates in statistical models, by performing 1,000 Montecarlo simulations to construct a confidence envelope of a complete spatial random distribution of the data and infer the existence and type of spatial trend. We thus performed a Detrended Correspondence Analysis (DCA) on the quantitative species composition, to evaluate patterns of similarity among our contrasted surveyed landscapes. Semivariograms were executed in the “geoR” package [71] in R [72].

We proceeded with a model selection approach to evaluate the type of relationship (linear or non-linear) that best explains responses of species richness and abundance to forest cover amount. Specifically, we ran separate model comparisons for the following groups of species: (*i*) overall community, (*ii*) the most common families (Fabaceae, Myrtaceae, Sapotaceae, Rubiaceae and Lauraceae), and (*iii)* each functional trait (regeneration strategy, dispersal mode, and seed size). For each of these groups, we tested four models: (1) null (constant); (2) linear; and two non-linear, (3) power law [73], and (4) four-parameter logistic [74]. The four-parameter logistic model has been widely used to investigate threshold relationships in fragmented tropical forest landscapes [21,75]. When the four-parameter logistic was selected as the best fit model we calculated the threshold value. The four parameters control the upper and lower asymptotes, the velocity in which the model changes between asymptotes, and the inflection point, which represents the amount of forest cover nearby the rapid change in forest richness and composition values [11]. For all variables exhibiting spatial correlation, we performed spatial linear models (SLMs), which account for spatial autocorrelation [76]. In those cases, we tested only three models, because SLMs analyses unsuccessfully adjust logistic models, so this option was excluded from the model selection approach. In all cases, we determined the parsimonious models by selecting all those showing dAIC (delta Akaike Information Criterion) value ≤ 2.00 [77]. We built routines to adjust non-linear model parameters, minimizing the model’s log-likelihood using numerical optimization [78], wAICc (weight Akaike Information Criterion corrected) and confidence intervals. During our model parameterization procedure, we excluded those models that did not show convergent solutions during numerical optimization. In all those cases in which the null model appeared amongst the parsimonious models, we assumed that no other model best explained the specific pattern than the chance. All analyses were implemented in R [72] using the "bbmle", "nlme", "spdep" and "lme4" packages [73,79].

Given that a great number of studies highlighted the role of edge effect as a local driver of changes in tree diversity, composition and functioning in tropical fragmented landscapes [80,81], we also performed analysis including the average linear distance from all five forest-plots to the nearest edge. For this, we performed Generalized Linear Models (GLMs), Generalized Additive Models (GAMs) or SLMs, according to the forest cover relationship type, and thus examined the effects of forest cover and edge distance to each community attribute investigated in our study. We ran GLMs if the linear model was a parsimonious model, GAMs were used when non-linear models were found to be most parsimonious, or SLMs when the variable showed spatial correlation. We used the same model selection approach described above to test these different landscapes metrics. For each community attribute, we tested four models: the null, only forest cover, only forest edge, and forest cover plus forest edge. Forest cover and forest edge distance were log transformed. The analyses were implemented in R using the "MuMIn", "mgcv", " lmmfit" and "bbmle" packages [79,82].

## Results

We recorded a total of 1,955 individuals belonging to 512 species and 53 families across a total sampled area of 1 ha (divided equally among 20 sites). 80.07% of surveyed individuals were identified to species level (410 species), 15.23% to genus (78 species), and 4.68% to family level (24 species). We were able to assign regeneration strategies to 89.8% of surveyed species, dispersal strategies for 95.9% of surveyed species, and seed size for 66.3% of all zoochoric species. Of the 66% of species with seed size data, 48.1% came from published sources and 18.2% from direct measurement from field collected seeds (see classification on S1 Table). Across the three traits, we assigned trait values to 98.4% of surveyed individuals.

The number of individuals in each forest site ranged from 60 to 129 (mean ± SD, 98 ± 17.5), and the number of species varied from 32 to 85 (57 ± 14.2). The five common families considered separately in this study were the most species rich in our survey: Myrtaceae (84 species), Fabaceae (65), Rubiaceae (32), Sapotaceae (32), and Lauraceae (25; see S1 Table).

### Spatial correlation

Out of the fourteen response variables tested (richness and abundance of tree communities and species with specific functional traits), only three showed significant spatial correlation—overall community abundance, abundance of biotic-dispersed species and abundance of small-seeded species, with remnants located less than 30 km from other remnants being more similar to each other than expected by chance (see S1 Fig).

### Forest cover influence on tree communities

**Fig 1:**
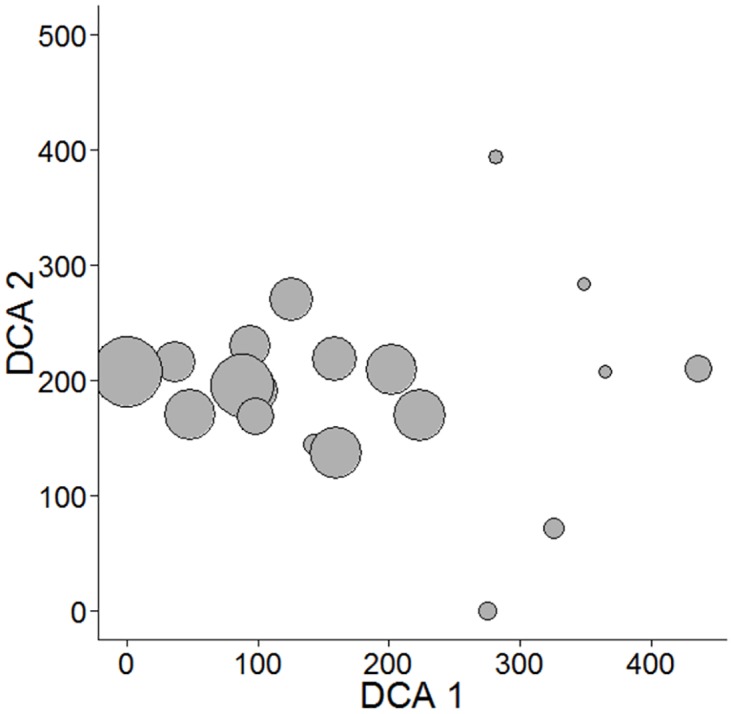
Detrended Correspondence Analysis (DCA) ordinations on the quantitative tree species composition.

**Fig 2:**
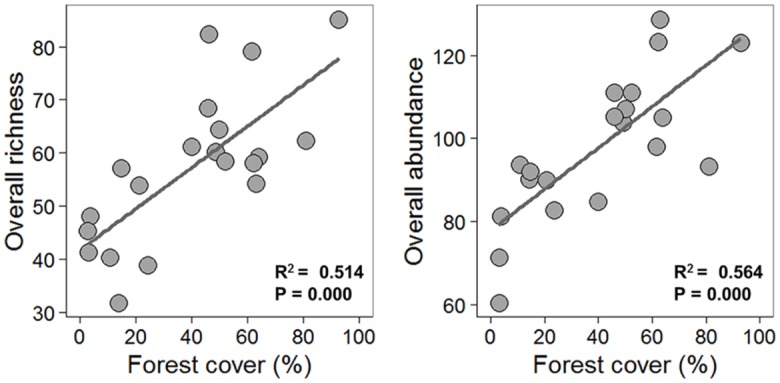
Relationships between forest cover amount with the overall species richness and overall abundance (number of individuals). Plotted curves represent all well-supported models (null, linear, power-law and logistic model) identified with AICc for each relationship. Logistic models were excluded for spatial linear models (see text for details).

**Fig 3:**
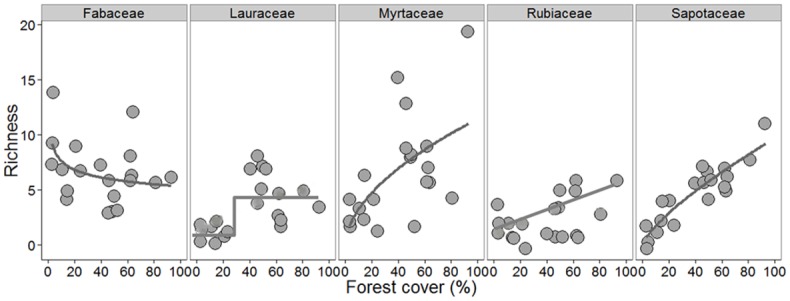
Relationships between forest cover with the species richness for each of the most common families recorded (Myrtaceae—Myr, Fabaceae—Fab, Lauraceae—Lau, Rubiaceae—Rub and Sapotaceae—Sap). Plotted curves represent all well-supported models (null, linear, power-law and logistic model) for each relationship.

The first DCA axis explained 59% of variance in community composition (*p* <0.001). Tree species composition was highly dissimilar in less forested landscapes (Fig 1). Overall, tree species richness and abundance were linearly related to forest cover amount, with our model predicting that every 10% reduction leads to a loss of 46 tree species and 83 individual trees. Non-linear models were also among the most parsimonious for species richness (Fig 2, S2 Table). At the family-level, we found a reduction in Myrtaceae and Sapotaceae species richness as forest cover decreased, within a 1000 m radius area, in linear and non-linear ways (linear and power law models selected), respectively (Fig 3). Lauraceae richness and abundance showed a sharp decrease with an inflection point of 29% (CI 95% 17.7–43.3%) of forest cover, based on the logistic model. Conversely, forest cover amount did not influence Rubiaceae and Fabaceae species richness (null model selected) (Fig 3, S2 Table).

**Fig 4:**
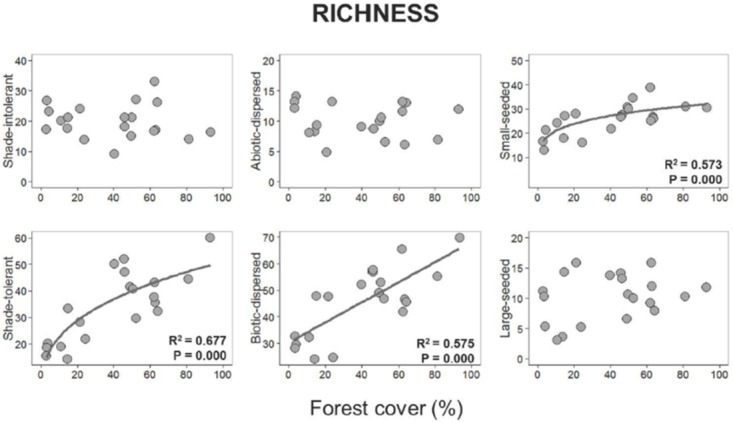
Relationships between forest cover amount with the species richness of shade-intolerant, shade-intolerance, abiotic-dispersed, biotic-dispersed, small-seeded and large-seeded species. Plotted curves represent of all well-supported models (null, linear, power-law and logistic model) identified with AICc for each relationship.

**Fig 5:**
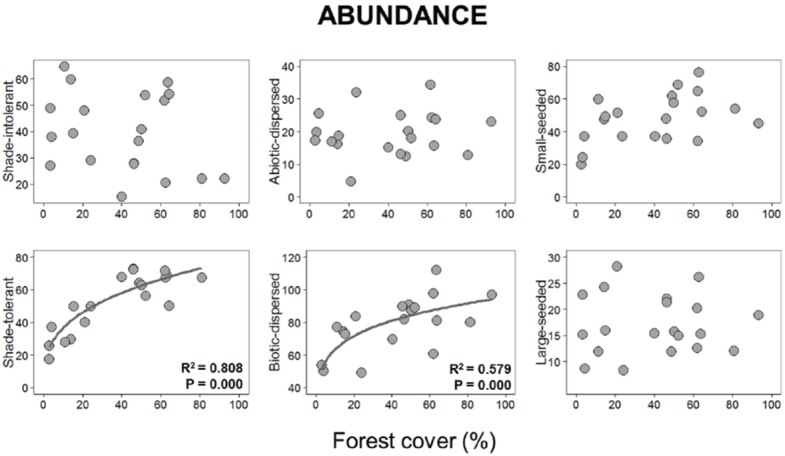
Relationships between forest cover amount with the abundance of shade-intolerant, shade-intolerance, abiotic-dispersed, biotic-dispersed, small-seeded and large-seeded species. Plotted curves represent of all well-supported models (null, linear, power-law and logistic model) identified with AICc for each relationship. Logistic models were excluded for spatial linear models (see text for details).

Considering the functional traits examined in this study, we found that forest cover reduction was associated with a decrease in shade-tolerant and biotic-dispersed species richness and abundance, with both linear and non-linear models explaining richness patterns, whereas the power-law best described the abundance patterns of those groups. Small-seeded species richness was also non-linearly affected by forest cover, with fewer of these species detected in sites surrounded by less forest (power law model selected). Forest cover did not affect the richness and abundance of shade-intolerant, abiotic-dispersed and large-seeded species and the abundance of small-seeded species, given that the null model was selected (Figs 4 and 5, S3 Table).

### Forest cover or edge distance?

When we conducted model selection analyses including forest cover amount (in a 1000 m radius) and nearest edge distance, we found that forest cover was the only variable included in the best model explaining the patterns for both overall community richness and abundance (S4 Table), with lower tree richness and abundance being recorded in forest sites within more deforested landscapes (Table 1). Likewise, at the family level, there were fewer Fabaceae species in remnants surrounded by lower amount of forest cover. The abundance of Fabaceae increased with forest cover reduction and edge proximity, whereas the species richness and abundance of Myrtaceae and Sapotaceae decreased only with forest cover reduction (Table 1 and S4 Table). By contrast, richness and abundance of Rubiaceae and Lauraceae were not associated with forest cover but were lower closer to forest edges (Table 1 and S4 Table).

**Table 1:** Coefficients and significance of the most parsimonious model (determined by AICc) for richness and abundance of the overall tree community and for the most important families in our study.

Parameter	Richness		Abundance	
	Edge distance	Cover amount	Edge distance	Cover amount
Overall^1^^,^^3^	+0.28	+0.23*	-	+4.16***
Myrtaceae^1^^,^^1^	-	+1.10***	-	+1.13***
Fabaceae^2^^,^^2^	-	NS	+14.78**	+41.81***
Lauraceae^2^^,^^2^	+6.33**	-	+7.02***	-
Rubiaceae^1^^,^^1^	+1.15*	-	+0.96*	-
Sapotaceae^1^^,^^1^	-	+1.71***	-	+1.82***

^1^GLM test (estimate);

^2^GAM test (Chi square value);

^3^SLM test (t-value).

The first and second number refers to the test used for species richness and abundance, respectively. When the null model was parsimonious we indicate using dash sign (-). Significance values: * p ≤0.05; ** p≤0.01;*** p≤0.001. NS: non-significant results.

For functional traits, the number of shade-tolerant, biotic-dispersed and small-seeded species was reduced in forest sites surrounded by lower amount of native forest, with this parameter alone included in the best model for these three trait classifications (Table 2 and S5 Table). Neither distance to edge nor percentage of forest cover were associated with the richness of shade-intolerant, large-seeded, and abiotic-dispersed species. For abundance patterns, the models containing only forest cover and forest cover plus edge distance were the most parsimonious for shade-tolerant and biotic-dispersed, whereas shade-intolerant, abiotic-dispersed, small- and large-seeded were not substantially associated with forest cover or distance to edge (Table 2 and S5 Table).

**Table 2:** Coefficients and significance of the best parsimonious model explaining patterns of species richness and abundance for each tree functional trait examined in our study.

Parameter	Richness		Abundance	
	Edge distance	Cover amount	Edge distance	Cover amount
Shade-intolerant^1^^,^^1^	-	-	-	NS
Shade-tolerant^1^^,^^2^	-	+0.78***	-	+109.5***
Biotic-dispersed^1^^,^^2^	-	+0.52***	NS	+17.58***
Abiotic-dispersed^1^^,^^3^	-	-	-	-
Small-seeded^2^^,^^3^	-	+16.72***	NS	-
Large-seeded^1^^,^^1^	-	NS	-	-

^1^GLM test (estimate);

^2^GAM test (Chi square value);

^3^SLM test (t-value).

The first and second number refers to the test used for species richness and abundance, respectively. When the null model was parsimonious we indicate using dash sign (-). Significance values: *** p≤0.001. NS: non-significant results

## Discussion

Our study demonstrates the powerful detrimental influence of landscape-scale forest cover loss on floristic diversity within Atlantic rainforest remnants. Specifically, we found that tree species richness and abundance, within remaining rainforest patches, significantly declined across a gradient of declining forest cover at the landscape scale. Additionally, our results suggest that tree communities persisting in severely deforested landscapes have experienced profound functional changes in tree community composition, with a directional loss in the number of shade-tolerant and biotically-dispersed species, and in the number of species within the most common families, such Myrtaceae and Sapotaceae, native to this tropical forest region.

### Overall tree community responses

Landscapes embedded within the highest deforestation levels showed the highest floristic differentiation among surveyed forest sites, evidence that deforested landscapes are maintaining high β-diversity [36]. Additionally, the amount of forest cover at the landscape-scale, a proxy for habitat amount, was linearly related to the overall richness and abundance of trees in the surveyed forest sites. Our knowledge regarding the role of landscape-scale forest cover affecting tree communities is still scarce, but few studies have consistently reported negative effects on species richness and abundance patterns [7,15,21,34,83]. We did not detect an extinction threshold for tree communities in our landscapes, using a 1000 m radius, as forest cover triggered a linear decay in both tree species richness and overall abundance. Indeed, extinction thresholds were less detectable at community level, since many species showing different ecological requirements were coupled together. Conversely, some studies have found a clear breakpoint in the relationship between forest cover and species richness when looking at families or functional traits [21,25,84].

Distance to forest edge at local scales was not a key predictor of overall tree species richness or abundance, a finding that contrasts with other studies in fragmented tropical forests (see [3,12,85]). Thus, our findings suggest that the amount of forest cover (1000 m radius) influenced the overall erosion of tree communities in anthropogenic landscapes, indicating a greater influence of habitat loss on the shaping of remaining tree communities. Our study therefore reinforces the importance of considering the amount of forest cover at landscape scales when accessing rainforest plant communities. As recent theory [17] and empirical studies support this conclusion [86], it is becoming increasingly clear that researchers should include this variable in future biodiversity studies in fragmented tropical landscapes. Thus conservation managers should consider this key landscape variable in management decisions for maintaining high diversity of flora assemblages in human modified landscapes.

### Tree family responses

When looking at dominant families individually, we also found a pronounced reduction in the species richness and abundance of common families along a gradient of decreasing forest cover within 1000m of survey sites. This pattern was strongest for Myrtaceae and Sapotaceae. These families are composed predominantly of shade-tolerant and zoochoric (animal dispersed) species, playing key roles for forest functionality in tropical forests [7,60,66]. The observed response patterns for Myrtaceae and Sapotaceae were quite similar to those for the overall tree community, that is, a strong decline along the forest cover gradient. In contrast, Fabaceae species richness was not affected by changes in forest cover amount, which may not be surprising given that this family includes both shade-tolerant and shade-intolerant species, many of which are known disturbance affiliates. Thus, the opposing response of these contrasting groups is likely to underlie the overall lack of response in this family. For Rubiaceae and Lauraceae, the proximity to forest edge seems to be an important factor leading to a decline in species richness and abundance. It probably indicates that these families are more susceptible to the effects of microenvironmental changes induced by edge effects.

An extinction threshold (around 29% forest cover) was only evident for Lauraceae in our study, despite past evidence of similar thresholds for Myrtaceae and Sapotaceae around 30% forest cover to adults trees in other Atlantic Forest landscapes [7,21]. These families are major sources of fleshy fruits in Atlantic forests, most providing important food resources for the native fauna [66,87]. Thus, declines of such species may lead to reduced resources for many vertebrate species persisting in forest remnants, and perhaps driving extinction cascades across trophic levels. This is further supported by past evidence of reductions in fruit biomass previously recorded in our study area [29].

### Functional traits responses

We detected an unequal decline of functional traits along gradients of forest cover amount in a 1000 m radius, but not to forest edge distance. Interestingly, shade-tolerant species were among the most vulnerable group of trees examined in our study, with both species richness and abundance of this group declining with reduced forest cover. Shade-tolerant trees are among the most common species in well preserved tropical rainforests, comprising ~98% of all species in Amazonian old-growth forests [6]. Indeed, the shade-tolerant group, which is mainly composed of late-successional species with high wood densities and higher maximum heights, is known to be more sensitive to anthropogenic changes at local (forest structure) and large-scales (landscape disturbance) than shade-intolerant species [62,88]. Our results showed that the abundance of shade-intolerant species was maintained along our forest cover gradient, a surprising outcome as we expected a compensatory effect in which sensitive groups (including shade-tolerant species) were replaced by many individuals of favored groups, as found previously in other studies [37,80]. We also did not observe a proliferation of disturbance-loving pioneer species, as reported in other Amazonian [6,85] and Atlantic forest fragmented landscapes [9,66]. However, due to the drastic reduction of shade-tolerant species in more deforested areas, the remaining species are mainly represented by disturbance affiliated species. Land-use history may be an important factor explaining the observed patterns in this study. For instance, unlike many regions of the Atlantic rainforest in Brazil, our landscapes were cleared quite recently (late twentieth century [89]) and do not have an extensive history of slash-and-burn agriculture. Such distinct long-term land use histories may lead to differences, for instance, in the extinction debts observed among regions.

Forest cover loss at landscape scale triggered a significant reduction of biotic-dispersed species reduction but not to forest edge distance. This group has previously been shown to be highly vulnerable to fragmentation and deforestation given its dependence on animal vectors for seed dispersal [90]. In extensively deforested landscapes, isolation among fragments generally increases, and such spatial disruptions of landscape structure lead to decreases in the likelihood of biotic dispersion among fragments [90,91]—a situation that might be aggravated in our region where large dispersers have been driven to near or complete extinction [46,92]. However, forest sites embedded within shade-cocoa matrix plantations can obtain higher turnover of animal dispersers, given that agroforestry mosaics enhance habitat availability and forest connectivity between forest sites [46]. The reduction in biotic-dispersed and shade-tolerant species, important resources for frugivorous birds and mammals [30,67,90], may have important consequences for forest dynamics. For instance, the loss of these species may drive changes to plant-animal interactions resulting in disruptions of vital functional services, such as seed dispersal and regeneration [93–95]. In contrast, abiotic-dispersed species were not affected by forest cover amount (at least within a 1000m radius) or forest edge distance. The similar distribution of these species across our forest sites suggests that wind and autochory dispersal may not be strongly influenced by the extent of forest within the landscape. Trees in deforested landscapes, as well as in small fragments, are likely to be more susceptible to wind action [96]. While such conditions can increase dispersal distances by wind, seeds dispersed beyond forest patches can be limited by a lack of suitable establishment and recruitment sites in the surrounding matrix. In temperate forests, where wind action is stronger, past studies have found that reduction of forest cover amount across landscapes is negatively related to species richness of wind-dispersed species [34]. Conversely, no effect was detected for this group within a highly fragmented tropical landscape in northeastern Brazil [9,66,97].

Surprisingly, we found that small-seeded species also declined in terms of species richness (but not in abundance) as forest cover amount declined. Conversely, the presence of large-seeded species was constant along the forest cover gradient. In our study area, large-seeded species represented 23% (± 18%) and 17% (± 12%) of the community species and abundance, respectively. These values are similar to the ones previously recorded in 31 Atlantic forest patches, where 21% of the tree species had large seeds [32]. Some of the large-seeded species are still found in deforested fragments, including *Virola gardneri*, *V*. *officinalis*, and *Pouteria bangii*. However, the presence of adults of these species may reflect an extinction debit, in which the reproduction and establishment of such species might be compromised by new environmental conditions, while adults are able to persist having established under previously more amenable conditions. Extinction debt was not evident in patterns of species richness of herbaceous vegetation in savanna [98]. Herbaceous or young vegetation, however, often responds faster to forest cover loss than tropical arboreal species, for which extinction debts are expected [7]. Our results suggest that the dispersal process has been hampered by reductions in forest cover amount across Brazil’s Atlantic forests, including the dispersal of small-seeded species, a pattern that is likely reinforced by the decline of frugivorous birds [25] and very low densities of large and medium mammals in the study region [46,92]. This latter demise is regionally attributed to a high and widespread hunting pressure [92], a process that is further aggravated by overall forest loss that is an important driver of defaunation [99].

## Conclusion

The change in species composition recorded in this study indicates that species loss is highly selective, with greater directional reduction with decreasing forest cover within a 1000m area in the number of shade-tolerant, biotic-dispersed and small-seeded species than shade intolerant, abiotically dispersed, and large-seeded species. Such floristic changes also show that the loss of shade-tolerant species is not being offset by increased numbers of shade-intolerant species. This suggests that Atlantic forest remnants in fragmented landscapes are changing in structural and compositional ways, linearly related to forest cover amount, whereas the distance to the nearest edge at landscape scales seems to play a secondary role, affecting only some families such as Rubiaceae and Lauraceae. Changes in specific functional traits suggest further negative impacts on the frugivorous animals that rely on these plant communities [66], while reductions in abundance is likely to decrease the capacity of these forest remnants to store carbon in the future [100]. Species changes may just be the beginning, reflecting initial shifts that will drive more extreme long-term modifications of forest pattern and process to other organisms and ecological functions. For instance, the declines in frugivorous-bird abundance and richness that have been recorded in our study area, may, in part, be driven by a lack of fruit producing trees in these rainforest remnants [25,29]. Likewise, increases in the rate of seed predation by invertebrates of an important palm species (*Euterpe edulis*) [101] may result from changes in tree diversity in remnants, something which may drive even greater changes to forest structure in the future [11]. These changes can lead to forest dynamics and ecosystem service disruptions, such as dispersal and pollination services. This study therefore highlights the importance of maintaining forest cover at landscape-scales as a means to prevent further changes in tree composition that are likely to drive cascading effects on the total biodiversity and ecosystem functioning of this important tropical region.

## Supporting information

S1 Fig Spatial correlation of species richness and abundance patterns. (PDF)

S1 Table Frequency of tree species and their respective classification on regeneration strategy, dispersion mode, and seed size, within 20 remnants with different forest cover amount in northeastern Brazil. (PDF)

S2 Table Ranking selection of best models explaining species richness and abundance of trees in function of forest cover amount at landscape-scale, for overall community and for the most important families. (PDF)

S3 Table Ranking selection of best models explaining species richness and abundance of trees in function of forest cover amount at landscape-scale, for each functional trait. (PDF)

S4 Table Ranking selection of best models explaining the richness and abundance of overall community and the most important families recorded for this study in function of forest cover amount and distance of nearest edge. (PDF)

S5 Table Ranking selection of best models explaining the richness and abundance of each functional trait in function of forest cover amount and distance of nearest edge. (PDF)
