# Supplementary material for: Functional decay in tree community within tropical fragmented landscapes: Effects of landscape-scale forest cover
Source: PLoS One. 2017 Apr 12;12(4):e0175545. doi: 10.1371/journal.pone.0175545 (PMC5389823; doi:10.1371/journal.pone.0175545)

Supporting Information

Functional decay in tree community within tropical fragmented landscapes: effects of landscape-scale forest cover

Larissa Rocha-Santos, Maíra Benchimol, Margaret Mayfield, Deborah Faria, Michaele Pessoa, Daniela Talora, Eduardo Mariano-Neto, Eliana Cazetta

S1 Fig - Spatial correlation of species richness and abundance patterns. Semivariograms of variables with spatial dependence: abundance of overall community, biotic-dispersed species and of small-seeded species. On the bottom, circles are sized proportional to the number of individuals in each landscape.

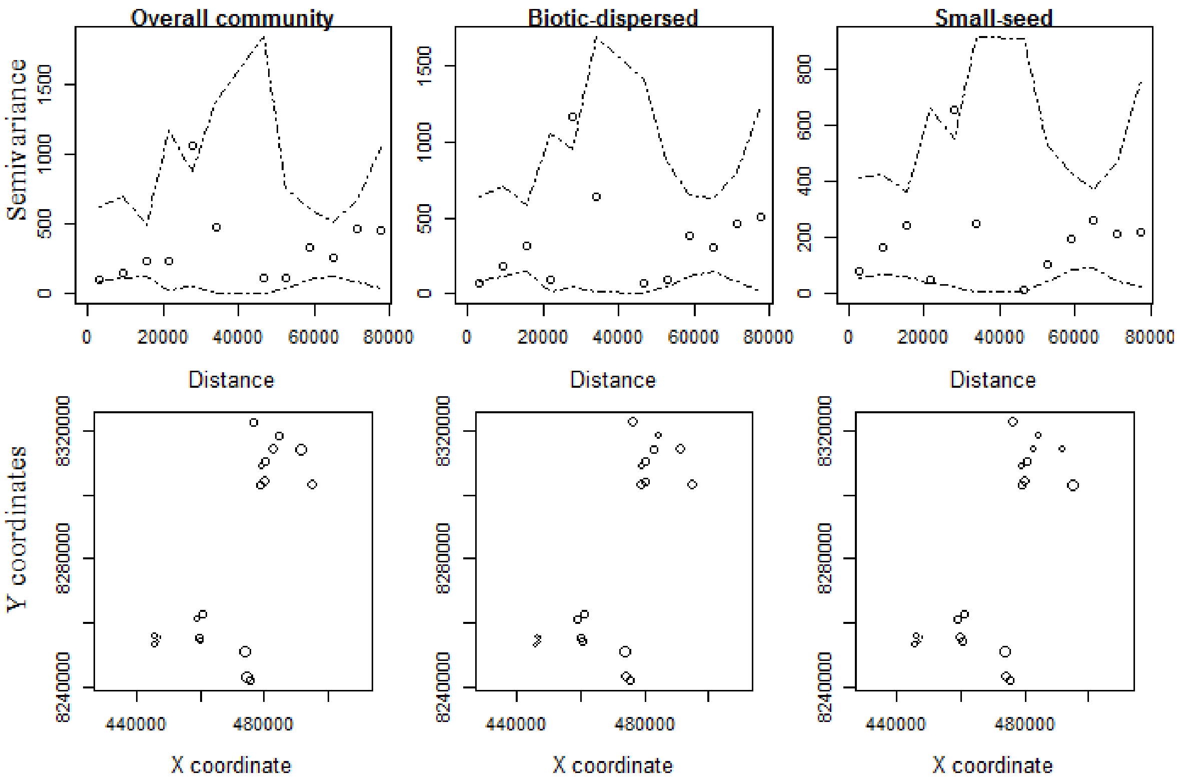

Supplement: S1 Fig — (PDF) [file pone.0175545.s001.pdf]
