# Supplementary material for: Functional decay in tree community within tropical fragmented landscapes: Effects of landscape-scale forest cover
Source: PLoS One. 2017 Apr 12;12(4):e0175545. doi: 10.1371/journal.pone.0175545 (PMC5389823; doi:10.1371/journal.pone.0175545)
Supplement: S1 Table — (PDF) [file pone.0175545.s002.pdf]

Supporting Information

Functional decay in tree community within tropical fragmented landscapes: effects of landscape-scale forest cover

Larissa Rocha-Santos, Maíra Benchimol, Margaret Mayfield, Deborah Faria, Michaele Pessoa, Daniela Talora, Eduardo Mariano-Neto, Eliana Cazetta

S1 Table - Frequency of tree species and their respective classification on regeneration strategy, dispersion mode, and seed size, within 20 forest sites with different forest cover amount in northeastern Brazil.

FAMILIES AND SPECIES	Regen.	Disp.	Seed	Nº ind.
**Achariaceae**				**5**
Carpotroche brasiliensis	ST	BI	LS	5
**Anacardiaceae**				**51**
Astronium graveolens	ST	AB	NC	4
Tapirira guianensis	SI	BI	SS	37
Thyrsodium spruceanum	SI	BI	SS	10
**Annonaceae**				**55**
Annona cacans	SI	BI	SS	2
Annona glabra	ST	BI	SS	3
Annona leptopetala	SI	BI	SS	3
Annona muricata	ST	BI	NC	2
Annona neolaurifolia	SI	BI	SS	10
Annona salzmannii	SI	BI	SS	1
Annona sylvatica	NC	BI	NC	2
Annonaceae 5	NC	BI	NC	1
Guatteria australis	SI	BI	SS	8
Guatteria oligocarpa	SI	BI	SS	3
Guatteria pogonopus	SI	BI	SS	2
Hornschuchia obliqua	SI	BI	NC	2
Pseudoxandra bahiensis	SI	BI	NC	3
Rollinia sp1	NC	BI	NC	1
Rollinia sp2	NC	BI	NC	1
Xylopia aromatica	SI	BI	SS	6
Xylopia frutescens	SI	BI	SS	1
Xylopia ochrantha	SI	BI	NC	1
Xylopia sericea	SI	BI	SS	3

**Apocynaceae**				**44**
Aspidosperma discolor	ST	AB	NC	6
Aspidosperma illustre	ST	AB	NC	1
Aspidosperma parvifolium	ST	AB	NC	2
Aspidosperma spruceanum	ST	AB	NC	1
Geissospermum laeve	SI	BI	SS	2
Himatanthus articulatus	SI	AB	NC	2
Himatanthus bracteatus	SI	AB	NC	14
Himatanthus phagedaenicus	SI	AB	NC	1
Lacmellea bahiensis	ST	BI	SS	5
Macoubea guianensis	SI	BI	SS	1
Malouetia cestroides	SI	NC	NC	2
Rauvolfia grandiflora	SI	BI	SS	3
Tabernaemontana brasiliensis	SI	BI	NC	1
Tabernaemontana salzmannii	ST	BI	SS	3
**Araliaceae**				**16**
Schefflera morototoni	SI	BI	SS	16
**Arecaceae**				**111**
Allagoptera caudescens	SI	BI	LS	35
Bactris ferruginea	SI	BI	LS	3
Bactris setosa	SI	BI	LS	1
Euterpe edulis	ST	BI	SS	51
Syagrus botryophora	SI	BI	LS	21
**Bignoniaceae**				**17**
Bignoniaceae 1	NC	AB	NC	1
Handroanthus chrysotrichus	ST	AB	NC	4
Handroanthus impetiginosus	SI	AB	NC	4
Jacaranda jasminoides	ST	AB	NC	1
Jacaranda puberula	ST	AB	NC	1
Jacaranda sp2	NC	AB	NC	2
Tabebuia cassinoides	SI	AB	NC	1
Tabebuia elliptica	SI	AB	NC	3
**Boraginaceae**				**30**
Cordia anabaptista	SI	BI	SS	2
Cordia ecalyculata	SI	BI	SS	15
Cordia glabrifolia	SI	BI	NC	1
Cordia membranacea	SI	BI	NC	2
Cordia mucronata	SI	BI	NC	1
Cordia polycephala	SI	BI	SS	1
Cordia sp3	SI	BI	NC	1
Cordia taguahyensis	SI	BI	SS	2
Cordia toqueve	SI	BI	SS	4

Cordia trachyphylla	SI	BI	NC	1
**Burseraceae**				**35**
Protium aracouchini	ST	BI	SS	4
Protium heptaphyllum	ST	BI	SS	18
Protium icicariba	ST	BI	NC	3
Protium sp1	ST	BI	NC	1
Protium warmingiana	ST	BI	SS	6
Tetragastris catuaba	ST	BI	NC	3
**Calophyllaceae**				**1**
Kielmeyera elata	ST	NC	NC	1
**Caricaceae**				1
Jacaratia heptaphylla	SI	BI	SS	1
**Caryocaraceae**				1
Caryocar edule	*ST*	BI	LS	1
**Celastraceae**				**9**
Cheiloclinium cognatum	ST	BI	LS	3
Maytenus distichophylla	SI	BI	SS	2
Maytenus obtusifolia	ST	BI	NC	1
Maytenus patens	SI	BI	NC	2
Tontelea mauritioides	ST	BI	NC	1
**Chrysobalanaceae**				**27**
Couepia belemii	ST	BI	NC	3
Couepia impressa	ST	BI	NC	2
Couepia monteclarensis	ST	BI	NC	3
Hirtella hebeclada	SI	BI	SS	1
Licania discolor	ST	BI	SS	1
Licania hoehnei	ST	BI	NC	1
Licania lamentanda	ST	BI	LS	5
Licania littoralis	ST	BI	SS	1
Licania naviculistipula	ST	BI	NC	1
Parinari alvimii	ST	BI	SS	5
Parinari sp1	ST	BI	NC	4
**Clusiaceae**				**18**
Garcinia gardneriana	SI	BI	SS	1
Garcinia macrophylla	SI	BI	LS	5
Symphonia globulifera	SI	BI	LS	2
Tovomita brevistaminea	ST	BI	NC	1
Tovomita choisyana	ST	BI	NC	2
Tovomita mangle	ST	BI	SS	7
**Combretaceae**				**4**
Buchenavia sp1	NC	BI	NC	1
Combretum sp1	NC	BI	SS	1

Terminalia brasiliensis	ST	AB	NC	2
**Dichapetalaceae**				**3**
Stephanopodium blanchetianum	ST	BI	SS	2
Stephanopodium organense	ST	BI	NC	1
**Ebenaceae**				1
Diospyros sp1	SI	BI	NC	1
**Elaeocarpaceae**				**8**
Sloanea guianensis	ST	BI	SS	6
Sloanea obtusifolia	ST	BI	SS	1
Sloanea sp1	ST	BI	NC	1
**Erythroxylaceae**				**2**
Erythroxylum martii	SI	BI	SS	1
Erythroxylum squamatum	SI	BI	SS	1
**Euphorbiaceae**				**62**
Actinostemon appendiculatus	ST	AB	NC	4
Actinostemon verticillatus	ST	AB	NC	2
Alchornea glandulosa	SI	BI	SS	1
Alchornea triplinervia	ST	BI	SS	3
Aparisthmium cordatum	SI	AB	NC	3
Croton floribundus	SI	AB	NC	3
Croton macrobothrys	SI	AB	NC	3
Euphorbiaceae 2	NC	NC	NC	2
Euphorbiaceae 4	NC	NC	NC	1
Euphorbiaceae 6	NC	NC	NC	1
Euphorbiaceae 7	NC	NC	NC	3
Glycydendron amazonicum	SI	BI	SS	4
Hevea brasiliensis	SI	AB	NC	5
Mabea brasiliensis	SI	AB	NC	4
Mabea piriri	SI	AB	NC	4
Ophthalmoblapton crassipes	ST	BI	NC	5
Ophthalmoblapton pedunculare	ST	BI	SS	1
Pausandra megalophylla	ST	AB	NC	1
Sapium glandulosum	ST	AB	NC	3
Sebastiania gaudichaudii	SI	AB	NC	2
Sebastiania jacobinensis	SI	AB	NC	1
Senefeldera multiflora	ST	AB	NC	5
Tetrorchidium rubrivenium	SI	BI	SS	1
**Fabaceae**				**234**
Abarema turbinata	NC	NC	NC	1
Aeschynomene sensitiva	NC	NC	NC	1
Albizia pedicellaris	SI	AB	NC	9
Andira anthelmia	ST	BI	LS	1

Andira fraxinifolia	SI	BI	LS	1
Andira legalis	ST	BI	LS	1
Andira lewisii	ST	BI	LS	1
Andira marauensis	ST	NC	NC	2
Andira sp1	NC	NC	NC	1
Andira sp2	NC	NC	NC	1
Andira sp3	NC	NC	NC	1
Arapatiella emarginata	ST	AB	NC	1
Arapatiella psilophylla	ST	AB	NC	14
Bauhinia sp1	NC	AB	NC	2
Bowdichia virgilioides	ST	AB	NC	2
Calliandra calycina	ST	AB	NC	1
Chamaecrista amorimii	ST	AB	NC	2
Chamaecrista bahiae	ST	AB	NC	9
Chamaecrista duartei	ST	AB	NC	9
Copaifera langsdorffii	ST	BI	LS	2
Copaifera trapezifolia	ST	BI	LS	2
Dialium guianense	ST	BI	SS	16
Diplotropis incexis	SI	AB	NC	1
Dipteryx sp1	NC	AB	NC	1
Enterolobium monjollo	SI	AB	NC	2
Exostyles venusta	ST	AB	NC	1
Hymenaea aurea	ST	BI	NC	1
Hymenaea oblongifolia	ST	BI	SS	2
Hymenolobium janeirense	SI	NC	NC	1
Inga capitata	SI	BI	SS	4
Inga cylindrica	SI	BI	SS	2
Inga luschnathiana	SI	BI	NC	1
Inga sp1	SI	BI	NC	1
Inga sp2	SI	BI	NC	3
Inga sp3	SI	BI	NC	1
Inga striata	SI	BI	SS	4
Inga thibaudiana	SI	BI	LS	8
Inga vera	SI	BI	SS	21
Machaerium hirtum	SI	AB	NC	1
Macrolobium latifolium	ST	AB	NC	7
Melanoxylon brauna	ST	AB	NC	5
Mimosa sp1	NC	AB	NC	1
Moldenhawera blanchetiana	ST	AB	NC	4
Moldenhawera floribunda	ST	AB	NC	2
Parkia pendula	ST	AB	NC	5
Peltogyne confertiflora	SI	AB	NC	14
Peltogyne sp1	ST	AB	NC	2

Piptadenia gonoacantha	SI	AB	NC	3
Piptadenia paniculata	SI	AB	NC	3
Plathymenia foliolosa	ST	AB	NC	5
Poincianella pluviosa	SI	AB	NC	5
Pterocarpus rohrii	ST	AB	NC	9
Senegalia sp1	NC	AB	NC	4
Senna multijuga	SI	AB	NC	3
Stryphnodendron pulcherrimum	SI	AB	NC	4
Swartzia macrostachya	ST	BI	LS	1
Swartzia oblata	ST	BI	LS	2
Swartzia polita	ST	BI	LS	4
Swartzia simplex	ST	BI	LS	3
Tachigali densiflora	SI	AB	NC	4
Tachigali paratyensis	SI	AB	NC	4
Vatairea heteroptera	ST	AB	NC	2
Vataireopsis araroba	ST	AB	NC	1
Zollernia glabra	ST	BI	SS	1
Zollernia latifolia	ST	BI	SS	1
**Humiriaceae**				**6**
Humiria balsamifera	ST	BI	NC	2
Schistostemon retusum	ST	BI	LS	2
Vantanea bahiaensis	SI	BI	SS	1
Vantanea compacta	ST	BI	SS	1
**Hypericaceae**				**3**
Vismia baccifera	SI	BI	SS	1
Vismia guianensis	SI	BI	SS	1
Vismia latifolia	SI	BI	SS	1
**Icacinaceae**				1
Emmotum nitens	ST	BI	SS	1
**Lauraceae**				**91**
Aniba intermedia	SI	BI	SS	5
Beilschmiedia linharensis	ST	BI	SS	1
Cryptocarya mandioccana	ST	BI	NC	3
Lauraceae sp2	NC	BI	NC	1
Lauraceae sp3	NC	BI	NC	1
Lauraceae sp4	NC	BI	NC	2
Licaria bahiana	SI	BI	SS	8
Licaria guianensis	SI	BI	LS	10
Nectandra membranacea	ST	BI	SS	11
Nectandra purpurea	ST	BI	NC	1
Nectandra sp1	NC	BI	NC	7
Ocotea aciphylla	ST	BI	LS	3

Ocotea cernua	ST	BI	NC	1
Ocotea divaricata	ST	BI	NC	5
Ocotea elegans	SI	BI	SS	5
Ocotea indecora	SI	BI	SS	5
Ocotea insignis	ST	BI	LS	1
Ocotea longifolia	SI	BI	SS	2
Ocotea macrophylla	ST	BI	NC	2
Ocotea montana	ST	BI	NC	1
Ocotea notata	ST	BI	NC	1
Ocotea oppositifolia	ST	BI	NC	4
Ocotea percoriacea	ST	BI	NC	2
Ocotea puberula	SI	BI	SS	8
Ocotea sp1	NC	BI	NC	1
**Lecythidaceae**				**62**
Eschweilera ovata	SI	BI	LS	50
Lecythis lurida	ST	BI	LS	5
Lecythis pisonis	ST	BI	LS	7
**Malpighiaceae**				**20**
Byrsonima alvimii	ST	BI	SS	2
Byrsonima fanshawei	ST	BI	NC	2
Byrsonima laxiflora	ST	BI	SS	1
Byrsonima sericea	SI	BI	SS	12
Byrsonima stipulacea	SI	BI	SS	3
**Malvaceae**				**65**
Apeiba albiflora	SI	BI	SS	1
Apeiba tibourbou	SI	AB	NC	4
Basiloxylon brasiliensis	SI	AB	NC	1
Ceiba sp1	NC	AB	NC	1
Eriotheca globosa	ST	AB	NC	5
Eriotheca macrophylla	ST	AB	NC	35
Hydrogaster trinervis	SI	AB	NC	1
Luehea divaricata	SI	AB	NC	4
Pavonia makoyana	ST	AB	NC	1
Pavonia morii	ST	AB	NC	2
Quararibea sp1	NC	BI	NC	1
Quararibea turbinata	SI	BI	LS	1
Sterculia apetala	ST	AB	NC	8
**Melastomataceae**				**59**
Henriettea succosa	SI	BI	SS	16
Melastomataceae 1	SI	BI	NC	1
Miconia amoena	SI	BI	SS	3
Miconia centrodesma	SI	BI	SS	6

Miconia dorsaliporosa	SI	BI	SS	4
Miconia hypoleuca	SI	BI	SS	7
Miconia minutiflora	SI	BI	SS	2
Miconia mirabilis	SI	BI	SS	3
Miconia prasina	SI	BI	SS	10
Miconia speciosa	SI	BI	SS	1
Tibouchina francavillana	SI	AB	NC	5
Tibouchina sp1	SI	AB	NC	1
**Meliaceae**				**21**
Guarea blanchetii	ST	BI	SS	7
Trichilia casaretti	ST	BI	SS	1
Trichilia elegans	ST	BI	SS	5
Trichilia lepidota	SI	BI	LS	4
Trichilia silvatica	ST	BI	SS	4
**Moraceae**				**145**
Artocarpus heterophyllus	SI	BI	LS	5
Brosimum guianense	SI	BI	SS	6
Brosimum rubescens	ST	BI	SS	13
Clarisia biflora	SI	BI	NC	3
Clarisia ilicifolia	SI	BI	SS	2
Ficus americana	ST	BI	NC	1
Ficus christianii	ST	BI	NC	2
Ficus hirsuta	SI	BI	SS	1
Ficus insipida	ST	BI	SS	2
Ficus sp1	NC	BI	NC	1
Helicostylis tomentosa	SI	BI	SS	99
Maclura tinctoria	ST	BI	SS	2
Sorocea bonplandii	ST	BI	SS	1
Sorocea guilleminiana	ST	BI	SS	7
**Myristicaceae**				**30**
Virola gardneri	ST	BI	LS	15
Virola officinalis	ST	BI	LS	15
**Myrtaceae**				**166**
Calyptranthes sp1	NC	BI	NC	1
Calyptranthes sp4	NC	BI	NC	1
Campomanesia dichotoma	SI	BI	SS	2
Campomanesia laurifolia	ST	BI	SS	3
Eugenia adenantha	SI	BI	NC	3
Eugenia astringens	ST	BI	NC	1
Eugenia ayacuchae	ST	BI	NC	3
Eugenia candolleana	ST	BI	SS	1
Eugenia excelsa	ST	BI	NC	3

Eugenia florida	ST	BI	SS	1
Eugenia itapemirimensis	ST	BI	SS	1
Eugenia lacistema	ST	BI	NC	1
Eugenia luschnathiana	ST	BI	SS	1
Eugenia magnifica	ST	BI	NC	8
Eugenia melanogyna	ST	BI	LS	3
Eugenia pauciflora	ST	BI	SS	1
Eugenia pisiformis	ST	BI	SS	1
Eugenia platyphylla	ST	BI	NC	4
Eugenia plicata	ST	BI	NC	1
Eugenia prasina	SI	BI	NC	3
Eugenia pruniformis	ST	BI	SS	2
Eugenia rostrata	ST	BI	NC	1
Eugenia schottiana	ST	BI	NC	7
Eugenia sp1	ST	BI	NC	1
Eugenia sp10	ST	BI	SS	5
Eugenia sp11	ST	BI	NC	1
Eugenia sp13	ST	BI	LS	1
Eugenia sp15	ST	BI	NC	1
Eugenia sp17	ST	BI	NC	1
Eugenia sp22	ST	BI	NC	1
Eugenia sp23	ST	BI	NC	2
Eugenia sp25	ST	BI	NC	1
Eugenia sp28	ST	BI	NC	1
Eugenia sp5	ST	BI	NC	1
Eugenia sp6	ST	BI	NC	1
Eugenia sp7	ST	BI	NC	1
Eugenia sp9	ST	BI	NC	1
Eugenia subterminalis	ST	BI	SS	3
Eugenia umbellata	ST	BI	SS	1
Gomidesia fenzliana	ST	BI	SS	1
Marlierea excoriata	ST	BI	NC	2
Marlierea obversa	ST	BI	SS	5
Marlierea racemosa	ST	BI	SS	1
Marlierea rufa	ST	BI	SS	3
Marlierea sp1	ST	BI	NC	1
Marlierea sp2	ST	BI	SS	1
Marlierea sp3	ST	BI	NC	1
Marlierea sp4	ST	BI	NC	1
Marlierea sucrei	ST	BI	SS	5
Myrcia anceps	ST	BI	NC	1
Myrcia calyptranthoides	ST	BI	NC	1
Myrcia carvalhoi	ST	BI	SS	1

Myrcia eximia	ST	BI	NC	1
Myrcia gigantea	ST	BI	SS	3
Myrcia pseudomarlierea	ST	BI	NC	1
Myrcia racemosa	ST	BI	SS	6
Myrcia sp1	ST	BI	NC	1
Myrcia sp11	ST	BI	NC	1
Myrcia sp12	ST	BI	NC	3
Myrcia sp14	ST	BI	NC	2
Myrcia sp18	ST	BI	NC	1
Myrcia sp20	ST	BI	NC	1
Myrcia sp24	ST	BI	NC	1
Myrcia sp25	ST	BI	NC	1
Myrcia sp27	ST	BI	NC	1
Myrcia sp28	ST	BI	NC	1
Myrcia sp3	ST	BI	NC	2
Myrcia sp9	ST	BI	NC	3
Myrcia splendens	SI	BI	SS	15
Myrcia sylvatica	SI	BI	SS	2
Myrcia vittoriana	ST	BI	SS	1
Myrciaria guaquiea	ST	BI	SS	2
Myrtaceae 15	ST	BI	NC	1
Myrtaceae 32	ST	BI	NC	1
Myrtaceae 34	ST	BI	NC	1
Myrtaceae 35	ST	BI	NC	1
Myrtaceae 5	ST	BI	NC	1
Neomitranthes langsdorfii	ST	BI	NC	2
Plinia callosa	ST	BI	NC	3
Plinia edulis	ST	BI	LS	1
Plinia grandifolia	ST	BI	SS	1
Plinia muricata	ST	BI	NC	2
Plinia rara	ST	BI	NC	2
Plinia sp1	ST	BI	NC	1
**Nyctaginaceae**				**102**
Guapira hirsuta	ST	BI	NC	11
Guapira laxiflora	ST	BI	SS	9
Guapira nitida	ST	BI	SS	56
Guapira obtusata	ST	BI	NC	1
Guapira opposita	ST	BI	SS	8
Guapira sp1	NC	BI	NC	1
Neea duckei	ST	BI	NC	2
Neea floribunda	ST	BI	SS	1
Neea macrophylla	ST	BI	SS	4
Neea madeirana	ST	BI	NC	2

Neea verticillata	ST	BI	NC	7
**Olacaceae**				**16**
Aptandra sp1	NC	NC	NC	1
Aptandra tubicina	ST	BI	NC	4
Heisteria ovata	ST	BI	NC	1
Heisteria perianthomega	ST	BI	SS	5
Heisteria sp2	NC	BI	LS	1
Schoepfia brasiliensis	ST	BI	NC	1
Tetrastilydium brasiliense	ST	BI	NC	2
Tetrastylidium grandifolium	ST	BI	LS	1
**Peraceae**				**32**
Chaetocarpus echinocarpus	SI		NC	1
Pera glabrata	SI		SS	5
Pogonophora schomburgkiana	SI		NC	26
**Phyllanthaceae**				**3**
Hyeronima alchorneoides	SI	BI	SS	2
Margaritaria nobilis	ST	BI	SS	1
**Picramniaceae**				**1**
Picramnia glazioviana	NC	NC	NC	1
**Polygonaceae**				**9**
Coccoloba arborescens	SI	BI	NC	1
Coccoloba bullata	ST	BI	NC	2
Coccoloba glaziovii	SI	BI	NC	1
Coccoloba oblonga	SI	BI	NC	1
Coccoloba rosea	ST	BI	SS	3
Coccoloba sp1	NC	BI	NC	1
**Primulaceae**				**2**
Myrsine coriacea	SI	BI	SS	1
Myrsine guianensis	SI	NC	NC	1
**Proteaceae**				**3**
Roupala sp1	NC	AB	NC	3
**Quiinaceae**				**1**
Quiina glaziovii	ST	BI	SS	1
**Rubiaceae**				**67**
Alseis floribunda	SI	AB	NC	1
Alseis latifolia	ST	AB	NC	1
Amaioua guianensis	ST	BI	SS	3
Bathysa mendoncaei	ST	BI	NC	4
Bathysa sp1	NC	BI	NC	1
Chomelia pedunculosa	ST	BI	SS	2
Cordiera bahiensis	ST	BI	NC	1
Cordiera sp1	NC	BI	NC	1

Coussarea carvalhoi	ST	BI	NC	5
Coussarea ilheotica	ST	BI	SS	4
Faramea biflora	ST	BI	SS	1
Guettarda platyphylla	ST	BI	NC	1
Guettarda sp1	NC	BI	NC	1
Guettarda viburnoides	ST	BI	SS	1
Ixora muelleri	ST	BI	SS	3
Palicourea blanchetiana	ST	BI	SS	1
Posoqueria latifolia	ST	BI	SS	2
Psychotria bahiensis	ST	BI	NC	1
Psychotria mapourioides	ST	BI	SS	9
Psychotria sp1	ST	BI	NC	1
Psychotria tenerior	ST	BI	NC	2
Psychotria vellosiana	ST	BI	SS	2
Randia nitida	ST	BI	NC	1
Rubiaceae 1	ST	BI	NC	2
Rubiaceae 2	ST	BI	NC	1
Rubiaceae 4	ST	BI	NC	3
Rubiaceae 6	ST	BI	NC	2
Rubiaceae 7	ST	BI	NC	1
Rudgea involucrata	ST	BI	SS	1
Rudgea pachyphylla	ST	BI	NC	1
Simira glaziovii	SI	AB	NC	4
Tocoyena bullata	ST	BI	NC	3
**Rutaceae**				**17**
Almeidea caerulea	ST	AB	NC	1
Conchocarpus macrophyllus	ST	NC	NC	1
Dictyoloma vandellianum	ST	AB	NC	1
Hortia arborea	ST	BI	SS	2
Pilocarpus grandiflorus	SI	AB	NC	1
Pilocarpus sp1	ST	AB	NC	1
Pilocarpus spicatus	ST	AB	NC	1
Rutaceae 1	NC	NC	NC	1
Zanthoxylum nemorale	ST	BI	NC	1
Zanthoxylum rhoifolium	SI	BI	SS	7
**Salicaceae**				**46**
Banara brasiliensis	SI	BI	SS	1
Casearia arborea	SI	BI	NC	3
Casearia bahiensis	ST	BI	NC	3
Casearia commersoniana	ST	BI	SS	25
Casearia silvestris	SI	BI	SS	9
Casearia sp1	NC	BI	NC	1

Macrothumia kuhlmannii	SI	BI	NC	2
Salicaceae 1	NC	BI	NC	1
Salicaceae 2	NC	BI	NC	1
**Sapindaceae**				**34**
Allophylus edulis	ST	BI	SS	7
Allophylus sericeus	SI	BI	SS	2
Cupania impressinervia	ST	BI	SS	2
Cupania oblongifolia	ST	BI	SS	1
Cupania racemosa	SI	BI	SS	1
Cupania rugosa	SI	BI	NC	2
Cupania scrobiculata	SI	BI	SS	1
Cupania sp2	NC	BI	SS	1
Cupania sp4	NC	BI	NC	2
Cupania tenuivalvis	ST	BI	NC	1
Cupania torta	ST	BI	NC	5
Matayba discolor	SI	BI	SS	1
Matayba guianensis	ST	BI	SS	1
Matayba juglandifolia	SI	BI	NC	1
Sapindaceae 1	NC	NC	NC	2
Scyphonychium multiflorum	ST	AB	NC	1
Talisia cerasina	SI	BI	SS	2
Talisia esculenta	ST	BI	SS	1
**Sapotaceae**				**129**
Chrysophyllum gonocarpum	ST	BI	SS	2
Chrysophyllum lucentifolium	ST	BI	NC	1
Chrysophyllum splendens	ST	BI	SS	13
Diploon cuspidatum	ST	BI	SS	3
Ecclinusa ramiflora	ST	BI	SS	22
Manilkara longifolia	ST	BI	SS	2
Manilkara maxima	ST	BI	SS	4
Manilkara multifida	ST	BI	NC	2
Manilkara salzmannii	ST	BI	SS	6
Micropholis crassipedicellata	ST	BI	SS	4
Micropholis gardneriana	ST	BI	SS	14
Micropholis guyanensis	ST	BI	SS	1
Pouteria atlantica	ST	BI	NC	2
Pouteria bangii	ST	BI	LS	12
Pouteria bapeba	ST	BI	SS	1
Pouteria bilocularis	ST	BI	NC	1
Pouteria grandiflora	ST	BI	LS	10
Pouteria guianensis	ST	BI	LS	1
Pouteria macahensis	ST	BI	NC	1

Pouteria macrophylla	ST	BI	SS	5
Pouteria microstrigosa	ST	BI	NC	1
Pouteria peduncularis	ST	BI	LS	2
Pouteria procera	ST	BI	SS	2
Pouteria reticulata	ST	BI	LS	4
Pouteria sp1	NC	BI	NC	1
Pouteria sp3	NC	BI	NC	1
Pouteria sp4	NC	BI	NC	2
Pouteria venosa	ST	BI	LS	2
Pradosia kuhlmannii	ST	BI	NC	1
Pradosia lactescens	ST	BI	SS	3
Pradosia sp2	ST	BI	NC	1
Sapotaceae sp3	ST	BI	NC	2
**Simaroubaceae**				**2**
Simarouba amara	ST	BI	SS	2
**Siparunaceae**				**9**
Siparuna guianensis	*ST*	BI	SS	9
**Solanaceae**				**4**
Aureliana fasciculata	SI	BI	SS	1
Solanum sooretamum	SI	BI	SS	1
Solanum sp2	NC	BI	SS	2
**Urticaceae**				**26**
Cecropia cecropiifolia	SI	BI	LS	1
Cecropia hololeuca	SI	BI	SS	2
Cecropia pachystachya	SI	BI	SS	2
Pourouma guianensis	SI	BI	SS	1
Pourouma mollis	SI	BI	SS	6
Pourouma velutina	SI	BI	SS	14
**Violaceae**				**48**
Paypayrola blanchetiana	ST	AB	NC	4
Paypayrola sp1	ST	AB	NC	1
Rinorea guianensis	ST	AB	NC	43
**Total**				1955

Legend: regeneration strategy (ST: shade-tolerant, SI: shade-intolerant), dispersal mode (AB: abiotic-dispersed, BI: biotic-dispersed) and seed size (SS: small-seeded, LS: large-seeded). NC: no classification.
