# Supplementary material for: Functional decay in tree community within tropical fragmented landscapes: Effects of landscape-scale forest cover
Source: PLoS One. 2017 Apr 12;12(4):e0175545. doi: 10.1371/journal.pone.0175545 (PMC5389823; doi:10.1371/journal.pone.0175545)
Supplement: S2 Table — (PDF) [file pone.0175545.s003.pdf]

Supporting Information

Functional decay in tree community within tropical fragmented landscapes: effects of landscape-scale forest cover

Larissa Rocha-Santos, Maíra Benchimol, Margaret Mayfield, Deborah Faria, Michaele Pessoa, Daniela Talora, Eduardo Mariano-Neto, Eliana Cazetta

S2 Table - Ranking selection of best models explaining species richness and abundance of trees in function of forest cover amount at landscape-scale, for overall community and for the most important families. Models that did not present convergence on the likelihood estimates were excluded from the model selection procedure. The most parsimonious models (linear – Li, logistic – Lo, null – Nu and power-law – Po) are highlight in gray.

Var.	Richness				Abundance			
	Model	dAICc	df	w_i	Model	dAICc	df	w_i
Ov	Li	0.0	3	0.6	Li	0.0	5	0.99
	Lo	1.7	4	0.2	Nu	15.2	4	0.00
	Po	2.0	3	0.2	Po	24.0	5	<0.001
	Nu	12.7	2	<0.001				
Mry	Po	0.0	3	0.52	Li	0.0	3	0.48
	Li	0.3	3	0.45	Po	0.7	3	0.35
	Nu	5.5	2	0.03	Lo	2.2	4	0.16
					Nu	7.4	2	0.01
Fab	Po	0.0	3	0.52	Nu	0.0	2	0.50
	Nu	0.9	2	0.32	Po	1.8	3	0.21
	Li	2.4	3	0.16	Li	2.0	3	0.19
					Lo	3.2	4	0.10
Lau	Lo	0.0	4	0.91	Lo	0.0	4	0.72
	Po	5.4	3	0.06	Po	3.0	3	0.16
	Li	7.3	3	0.02	Li	4.4	3	0.08
	Nu	10.3	2	0.01	Nu	5.5	2	0.05
Rub	Li	0.0	3	0.57	Li	0.0	3	0.33
	Nu	1.9	2	0.22	Lo	0.4	4	0.27
	Po	2.0	3	0.21	Nu	0.6	2	0.24
					Po	1.4	3	0.16
Sap	Po	0.0	3	0.56	Li	0.0	3	0.57
	Li	0.4	3	0.44	Po	0.5	3	0.43
	Nu	30.6	2	<0.001	Nu	24.8	2	<0.001

Legend: Variables (Var.); overall (Ov); Mrytaceae (Mry); Rubiaceae (Rub); Sapotaceae (Sap); Fabaceae (Fab); Lauraceae (lau); difference in AICc from the best model (dAIC); parameter number of the model (df); AICc weight (wi).
