# Supplementary material for: Functional decay in tree community within tropical fragmented landscapes: Effects of landscape-scale forest cover
Source: PLoS One. 2017 Apr 12;12(4):e0175545. doi: 10.1371/journal.pone.0175545 (PMC5389823; doi:10.1371/journal.pone.0175545)
Supplement: S3 Table — (PDF) [file pone.0175545.s004.pdf]

Supporting Information

Functional decay in tree community within tropical fragmented landscapes: effects of landscape-scale forest cover

Larissa Rocha-Santos, Maíra Benchimol, Margaret Mayfield, Deborah Faria, Michaele Pessoa, Daniela Talora, Eduardo Mariano-Neto, Eliana Cazetta

S3 Table - Ranking selection of best models explaining species richness and abundance of trees in function of forest cover amount at landscape-scale, for each functional trait. Models that did not present convergence on the likelihood estimates were excluded from the model selection procedure. The most parsimonious models (linear – Li, logistic – Lo, null – Nu and power-law – Po) are highlight in gray.

	**Richness**				**Abundance**			
Var.	Model	dAICc	df	w_i	Model	dAICc	df	w_i
Si	Nu	0.0	2	0.6	Nu	0.0	2	0.5
	Po	2.3	3	0.2	Li	1.3	3	0.3
	Li	2.5	3	0.2	Po	2.1	3	0.2
St	Po	0.0	3	0.5	Po	0.0	3	0.9
	Li	0.8	3	0.3	Li	3.6	3	0.1
	Lo	2.0	4	0.2	Nu	29.2	2	<0.001
	Nu	18.4	2	<0.001				
Ad	Nu	0.0	2	0.6	Nu	0.0	4	0.8
	Po	2.5	3	0.2	Li	3.4	5	0.2
	Li	2.8	3	0.1	Po	15.7	5	<0.001
	Lo	4.0	4	0.1				
Bd	Li	0.0	3	0.6	Po	0.0	3	0.8
	Po	0.8	3	0.4	Li	3.7	3	0.1
	Nu	15.4	2	<0.001	Lo	4.1	4	0.1
					Nu	12.9	2	0.0
Ls	Nu	0.0	2	0.4	Nu	0.0	2	0.6
	Po	0.7	3	0.3	Po	2.7	3	0.2
	Li	0.8	3	0.3	Li	2.8	3	0.2
					Lo	6.0	4	0.0
Ss	Po	0.0	3	0.6	Li	0.0	5	0.7
	Li	2.3	3	0.2	Nu	1.4	4	0.3
	Lo	2.8	4	0.2	Po	15.5	5	<0.001
	Nu	12.8	2	0.0				

Legend: Variable (Var.); shade-intolerant (Si); shade-tolerant (St); abiotic-dispersed (Ad); biotic-dispersed (Bd); large-seeded (Ls); small-seeded (Ss); difference in AICc from the best model (dAIC); parameter number of the model (df); AICc weight (w_i).
