# Supplementary material for: Functional decay in tree community within tropical fragmented landscapes: Effects of landscape-scale forest cover
Source: PLoS One. 2017 Apr 12;12(4):e0175545. doi: 10.1371/journal.pone.0175545 (PMC5389823; doi:10.1371/journal.pone.0175545)
Supplement: S4 Table — (PDF) [file pone.0175545.s005.pdf]

Supporting Information

Functional decay in tree community within tropical fragmented landscapes: effects of landscape-scale forest cover

Larissa Rocha-Santos, Maíra Benchimol, Margaret Mayfield, Deborah Faria, Michaele Pessoa, Daniela Talora, Eduardo Mariano-Neto, Eliana Cazetta

S4 Table - Ranking selection of best models explaining the richness and abundance of overall community and the most important families recorded for this study in function of forest cover amount and distance of nearest edge. The most parsimonious (Nu - null; Co - Forest cover; Ed - Forest edge distance; Co + Ed - Forest cover plus edge distance) models are shown in gray.

Var.	Models	RICHNESS			Models	ABUNDANCE		
		dAIC	df	w_i		dAIC	df	w_i
Ov [1,3]	Co + Ed	0.0	3.0	0.50	Co	0.0	5.0	0.78
	Co	1.0	2.0	0.30	Co + Ed	3.3	6.0	0.15
	Ed	1.9	2.0	0.20	Ed	4.7	5.0	0.07
	Nu	31.2	1.0	0.00	Nu	15.2	4.0	0.00
Mry [1,1]	Co	0.0	2.0	0.75	Co	0.0	2.0	0.74
	Co + Ed	2.5	3.0	0.22	Co + Ed	2.3	3.0	0.24
	Ed	6.1	2.0	0.04	Ed	7.6	2.0	0.02
	Nu	20.2	1.0	0.00	Nu	27.9	1.0	0.00
Fab [2,2]	Co	0.0	2.1	0.46	Co + Ed	0.0	12.1	0.99
	Nu	1.6	1.0	0.21	Co	10.0	9.4	0.01
	Co + Ed	1.9	6.1	0.18	Ed	27.4	7.5	0.00
	Ed	2.1	4.1	0.16	Nu	50.8	1.0	0.00
Lau [2,2]	Ed	0.0	7.3	0.56	Ed	0.0	8.0	0.68
	Co + Ed	1.7	8.2	0.24	Co + Ed	1.6	9.0	0.31
	Co	2.0	5.0	0.21	Co	8.4	6.2	0.01
	Nu	19.5	1.0	0.00	Nu	41.1	1.0	0.00
Rub [1,1]	Ed	0.0	2.0	0.63	Ed	0.0	2.0	0.62
	Co + Ed	2.3	3.0	0.20	Co + Ed	2.6	3.0	0.17
	Nu	3.9	1.0	0.09	Co	3.6	2.0	0.10
	Co	4.2	2.0	0.08	Nu	3.7	1.0	0.10
Sap [1,1]	Co	0.0	2.0	0.80	Co	0.0	2.0	0.80
	Co + Ed	2.8	3.0	0.20	Co + Ed	2.7	3.0	0.21
	Ed	10.8	2.0	0.00	Ed	18.6	2.0	0.00
	Nu	28.9	1.0	0.00	Nu	44.7	1.0	0.00

[1]GLMM test; [2]GAMM test; [3]SLM test. The first number refers to the test used for richness and the second for abundance. Values of difference in AICc from the best model (dAIC); parameter number of the model (df); AICc weight (wi).
