# Supplementary material for: Functional decay in tree community within tropical fragmented landscapes: Effects of landscape-scale forest cover
Source: PLoS One. 2017 Apr 12;12(4):e0175545. doi: 10.1371/journal.pone.0175545 (PMC5389823; doi:10.1371/journal.pone.0175545)
Supplement: S5 Table — (PDF) [file pone.0175545.s006.pdf]

Supporting Information

Functional decay in tree community within tropical fragmented landscapes: effects of landscape-scale forest cover

Larissa Rocha-Santos, Maíra Benchimol, Margaret Mayfield, Deborah Faria, Michaele Pessoa, Daniela Talora, Eduardo Mariano-Neto, Eliana Cazetta

S5 Table - Ranking selection of best models explaining the richness and abundance of each functional trait in function of forest cover amount and distance of nearest edge. The most parsimonious models (Nu - null; Co - Forest cover; Ed - Forest edge distance; Co + Ed - Forest cover plus edge distance) are shown in gray.

Var.	Models	RICHNESS			Models	ABUNDANCE		
		dAIC	df	w_i		dAIC	df	w_i
Si [1,1]	Nu	0.0	1.0	0.52	Co	0.0	2.0	0.41
	Co	1.8	2.0	0.21	Ed	1.0	2.0	0.25
	Ed	2.4	2.0	0.15	Nu	1.2	1.0	0.23
	Co + Ed	3.0	3.0	0.12	Co + Ed	2.7	3.0	0.11
St [1,2]	Co	0.0	2.0	0.77	Co	0.0	7.3	0.59
	Co + Ed	2.4	3.0	0.23	Co + Ed	0.7	12.7	0.41
	Ed	21.6	2.0	0.00	Ed	14.5	7.9	0.00
	Nu	65.6	1.0	0.00	Nu	116.5	1.0	0.00
Ad [1,3]	Nu	0.0	1.0	0.58	Nu	0.0	4.0	0.73
	Co	2.2	2.0	0.20	Co	3.4	5.0	0.13
	Ed	2.4	2.0	0.17	Ed	3.5	5.0	0.13
	Co +Ed	4.9	3.0	0.05	Co + Ed	7.6	6.0	0.02
Bd [1,2]	Co	0.0	2.0	0.55	Co + Ed	0.0	4.3	0.71
	Co + Ed	0.5	3.0	0.43	Co	1.8	5.0	0.29
	Ed	6.7	2.0	0.02	Ed	15.0	2.0	0.00
	Nu	40.6	1.0	0.00	Nu	49.8	1.0	0.00
Ls [1,1]	Co	0.0	2.0	0.36	Nu	0.0	1.0	0.59
	Nu	0.3	1.0	0.30	Ed	2.3	2.0	0.19
	Ed	0.7	2.0	0.25	Co	2.4	2.0	0.18
	Co + Ed	2.7	3.0	0.09	Co + Ed	5.1	3.0	0.05
Ss [2,3]	Co	0.0	2.0	0.41	Ed	0.0	5.0	0.38
	Co + Ed	0.2	3.1	0.36	Co	0.1	5.0	0.37
	Ed	1.1	2.0	0.23	Nu	1.5	4.0	0.19
	Nu	16.1	1.0	0.00	Co + Ed	3.6	6.0	0.06

[1]GLMM test; [2]GAMM test; [3]SLM test. The first number refers to the test used for richness and the second for abundance. Values of difference in AICc from the best model (dAIC); parameter number of the model (df); AICc weight (w_i).
